# Serum sodium in relation to various domains of cognitive function in the elderly US population

**DOI:** 10.1186/s12877-021-02260-4

**Published:** 2021-05-24

**Authors:** Sohyae Lee, Jin-young Min, Beom Kim, Sang-Won Ha, Jeohng Ho Han, Kyoung-bok Min

**Affiliations:** 1 Department of Preventive Medicine, College of Medicine, Seoul National University, Seoul, Republic of Korea; 2 Veterans Medical Research Institute, Veterans Health Service Medical Center, Seoul, Republic of Korea; 3 Division of Nephrology, Department of Internal Medicine, Veterans Health Service Medical Center, Seoul, Republic of Korea; 4 Department of Neurology, Veteran Healthcare Service Medical Center, Seoul, Republic of Korea; 5 Institute of Health Policy and Management, Medical Research Center, Seoul National University, Seoul, South Korea

**Keywords:** Serum sodium, Hyponatremia, Cognitive function, Elderly

## Abstract

**Background:**

Recent evidence suggests that sodium imbalances may be associated with cognitive impairment; however, the association between specific domains of cognition remains unclear. This study examines the association between serum sodium levels and immediate and delayed verbal memory as measured by the CERAD Word Learning Test (CERAD WLT), executive function as measured by the Animal Fluency test (AFT), and sustained attention, working memory, and processing speed as measured by the Digit Symbol Substitution test (DSST) in the elderly population of the US aged 60 and older who participated in the 2011–2014 National Health and Nutrition Examination Surveys (n = 2,541).

**Methods:**

Cognitive function tests were performed by trained interviewers and sodium levels were measured using indirect ion selective electrode methodology.

**Results:**

After adjusting for all covariates, quintiles of CERAD WLT scores showed significant positive associations with log-transformed sodium levels (Immediate recall (IR) β = 4.25 (SE = 1.83, p-value 0.027); Delayed recall (DR) β = 6.54 (SE = 1.82, p-value 0.001)). Compared to normal sodium levels, hyponatremia was significantly associated with lower CERAD WLT-IR (β = -0.34, SE = 0.15, p-value 0.035) and CERAD WLT-DR scores (β -0.48, SE = 0.10, p-value < 0.001) and showed borderline significance with AFT scores (β = = -0.38, SE = 0.19, p-value 0.052). Hypernatremia did not show any significant relationships with cognitive test scores, compared to normal sodium levels.

**Conclusions:**

Our cross-sectional study showed that lower sodium levels were associated with cognitive change, especially regarding memory and executive function.

## Background

Sodium is one of the major extra-cellular fluid electrolytes, which is important in maintaining extracellular fluid volume and potentials across cell membranes [1, 2]. Imbalances in sodium concentrations have been known to manifest as headaches, confusion, nausea, and restlessness, while rapid changes in sodium concentrations result in acute neurologic symptoms such as seizures and impaired mental status [2, 3]. Hyponatremia is commonly defined as serum sodium concentrations less than 135 mmol/L and is prevalent in the elderly due to impaired water-excretory capacity associated with normal aging [1, 4]. Most cases of hyponatremia are mild and relatively asymptomatic; however, recent evidence suggests that hyponatremia may be associated with gait disturbances, falls, and cognitive impairment [4–8].

Only a few studies have addressed the relationship between serum sodium levels and cognitive function; however, the definition of cognitive function appears to vary among studies. In addition, studies only examine single domains of cognitive function, or assess multiple domains grouped as a single variable. Previous studies addressing the relationship between serum sodium levels and cognitive function have assessed cognitive function using a combination of attention tests (Visual Vigilance, Working Memory or Digit Span, Go/No Go, Intermodal Comparison, Divided Attention, Phasic Alert tests) [7], the Audio Recorded Cognitive Screening (ARCS) tool [8], a combination of the modified Mini-Mental Status Exam (MMSE) and the Trail Making Test [9], and a combination of the MMSE and Clock Completion Test [5]. In addition, most previous studies are limited to specific populations (i.e. men or single hospital settings) [5, 7, 9].

Cognitive change is part of the normal process of aging [10]. In contrast to cognitive domains such as language, some cognitive abilities such as memory, executive function, and processing speed decline over time, and the rate of decline varies among individuals [10, 11]. Cognitive performance is usually categorized in terms of domains of functioning (i.e. executive functioning, processing speed), and these domains are linked to specific areas of the brain [12, 13]. In order to differentiate between the various types of conditions causing cognitive impairment, specific subdomains are assessed separately [13]. As a result, it is of importance to determine the specific domains of cognitive function associated with serum sodium levels. Our study aims to assess the relationship between serum sodium levels and various domains of cognitive function including, memory, executive function, and processing speed using the CERAD Word Learning Test (CERAD WLT), Animal Fluency test (AFT), and the Digit Symbol Substitution test (DSST) in the elderly population of the US aged 60 and older.

## Methods

### Study population

The National Health and Nutrition Examination Survey (NHANES) is a major program of the National Center for Health Statistics, part of the Centers for Disease Control and Prevention. The survey is designed to compile information on the health and nutritional status of adults and children in the US. The NHANES conducts interviews for demographic, socioeconomic, dietary, and health-related questions and physical examinations for medical, dental, and physiological measurements, as well as laboratory tests. The survey is administered by highly trained medical personnel [14].

We used the 2011–2012 and 2013–2014 National Health and Nutrition Examination Surveys (NHANES), which is a nationally representative sample of the non-institutionalized population institutionalized population in the US [14]. This study included a total of 19,931 individuals with data on serum sodium levels and cognitive test scores. All study participants aged 60 years and older (n = 3,632), who consented to the mobile examination center exam (n = 3,472), did not need a proxy informant and read and understood English, Spanish, Korean, Vietnamese, traditional or simplified Mandarin, or Cantonese were eligible for cognitive function testing (n = 3,398) [15, 16]. Among a total of 3,398 participants eligible for testing, CERAD WLT-IR was conducted in 3,131 individuals. Among these subjects, serum sodium levels were measured in 2,946 participants, and 2,541 participants had data for all covariates.

### Cognitive tests

The NHANES cognitive functioning questionnaire included the CERAD WLT, AFT, and DSST. The CERAD WLT evaluates immediate and delayed verbal memory. The test is composed of three consecutive learning trials, followed by a delayed recall test. Each learning trial is followed by an immediate recall test. In the learning trials, participants were presented with 10 words. Immediately following the learning trial, participants were instructed to recall as many words as possible. The number of words correctly recalled was recorded as the score for each learning trial, with a maximum possible score of 10. This process was repeated three times, and the final score for the CERAD WLT-IR was recorded as the sum of scores from the three trials. The delayed recall test was conducted after conducting the AFT and DSST (approximately 8–10 min after starting the CERAD WLT). Participants were asked to recall as many words as possible, and scores were recorded as a number out of 10. The AFT assesses executive function. Participants were instructed to name as many animals as possible in one minute, and the number of animals named was recorded as the score. Individuals who did not pass the practice test, naming three articles of clothing, did not participate in the AFT. The DSST examines sustained attention, working memory, and processing speed. The test consists of a paper form that has a legend at the top in which 9 numbers are matched with 9 symbols. Participants were given 133 numbers and were asked to copy the corresponding symbol presented in the legend. The total number of correct matches copied within 2 min was recorded as the score out of 133 [15, 16].

### Measurements of serum sodium levels

Blood samples were collected by a phlebotomist at the NHANES Mobile Examination Centers [17, 18]. Specimens were stored at appropriate refrigerated conditions (2–8 degrees Celsius) before being shipped to the Collaborative Laboratory Services, Ottumwa, Iowa for analysis. Sodium levels were measured using the DxC800 system using indirect ion selective electrode methodology. The DxC800 measures sodium levels using the voltage created by sodium ion exchange [19, 20].

### Other variables of interest

Other variables of interest included socio-demographic factors and health behaviors and conditions. Socio-demographic factors were age, sex, ethnicity (non-Hispanic white, non-Hispanic black, Hispanic, or other), annual household income (≤$20,000 or ≥$20,000), educational levels (less than high school, high school graduate, or more than high school), and marital status (married, never married, widowed, divorced, separated, or living with partner). Heath behavior variables included cigarette smoking status (never smoker, former smokers, or current smoker), alcohol consumption (yes or no), and moderate-intensity physical activity status (yes or no). Health conditions were referred to as body mass index, clinical examination, diuretic prescription, and medical history. BMI was calculated using person’s weight and height and categorized into four categories: underweight (< 18.5 kg/m2), normal weight (18.5–24.9 kg/m2), overweight (25.0–29.9 kg/m2), and obese (> 30 kg/m2). Clinical examination related variables were glomerular filtration rate (GFR) (ml/min per 1.73 m^2^) and serum glucose levels (mg/dL). GFR was calculate using the Chronic Kidney Disease Epidemiology Collaboration equation (CKD-EPI eGFR) [21]. Data on a history of cardiovascular disease (defined as a history of coronary heart disease, angina, or heart failure), diabetes, chronic pulmonary obstructive disease (COPD), thyroid disease, stroke, and liver disease was also included.

### Statistical analysis

Serum sodium levels were right–skewed and log transformed. Log-transformed serum sodium levels were analyzed as both continuous and categorical variables by hyponatremia, normonatremia, and hypernatremia status. Serum sodium below 135 mmol/L was classified as hyponatremia, and serum sodium above 145 mmol/L was classified as hypernatremia [1]. Cognitive tests scores were analyzed as quintiles because scores were not normally distributed. Correlation analysis was performed using Pearson’s correlation coefficients between cognitive test scores and serum sodium levels were calculated. Linear regression analysis was performed to determine the relationship between serum sodium levels and cognitive test scores. Beta coefficients and standard errors (SE) for cognitive test scores among individuals with hyponatremia and hypernatremia compared to those with the normal serum sodium levels as the reference group. The linear regression model was adjusted for all potential confounders including age, sex, race, income, education, marital status, smoking history, alcohol consumption, physical activity, BMI, GFR, serum glucose, diuretic use, and a history of cardiovascular disease, diabetes, COPD, thyroid disease, stroke, and liver disease.

All analyses were estimated by survey weight applied to complex sampling design [22] performed with the PROC SURVEY procedures of SAS 9.4 statistical analysis package (SAS Institute, Cary, NC, USA). A significant level was considered as α = 0.05.

## Results

The characteristics of the study population with data for CERAD WLT-IR (n = 2,541) are shown in Table 1. The mean age of participants was 69.6 (± 6.8) years (range: 60–80), and women represented 51.4 % of the overall sample. Most participants were non-Hispanic white (49.3 %), not impoverished (74.8 %), had an education beyond high school (51.0 %), and married (54.9 %). A majority of the participants were overweight (35.4 %) or obese (37.5 %). Mean CKD-EPI eGFR was 73.4 (± 19.8) ml/min per 1.73 m^2^ and mean serum glucose was 112.4 (± 43.0) mg/dL.

**Table 1:** Characteristics of the study population

Characteristics	Mean ± SD or N(%)
No of participants	2541
Sex	
Male	1236 (48.6 %)
Female	1305 (51.4 %)
Age at interview (year)	69.6 ± 6.8
Ethnicity	
Non-Hispanic white	1252 (49.3 %)
Non-Hispanic black	573 (22.6 %)
Hispanic	477 (18.8 %)
Others	239 (9.4 %)
Annual Family Income	
Less than $20,000	640 (25.2 %)
$20,000 and over	1901 (74.8 %)
Education	
Less than high school	645 (25.4 %)
High school graduate	600 (23.6 %)
More than high school	1296 (51.0 %)
Marital status	
Married	1395 (54.9 %)
Never married	146 (5.8 %)
Widowed/divorced/separated	1000 (39.4 %)
Smoking history	
Never smoked	329 (13.0 %)
Ex-Smoker	954 (37.5 %)
Current Smoker	1258 (49.5 %)
Alcohol consumption^a^	
Yes	1729 (68.0 %)
No	812 (32.0 %)
Physical Activity^b^	
Yes	985 (38.8 %)
No	1556 (61.2 %)
BMI (kg/m^2^)	
Underweight (< 18.5)	35 (1.4 %)
Normal weight (18.5–24.9)	653 (25.7 %)
Overweight (25.0-29.9)	899 (35.4 %)
Obesity (> 30)	954 (37.5 %)
CKD-EPI eGFR, ml/min per 1.73 m^2^	73.4 ± 19.8
Serum glucose, mg/dL	112.4 ± 43.0
Diuretic use	478 (18.8 %)
Diabetes	617 (24.3 %)
Cardiovascular disease^c^	383 (15.1 %)
Stroke	173 (6.8 %)
Chronic obstructive pulmonary disease	242 (9.5 %)
Thyroid disease	434 (17.1 %)
Liver disease	143 (5.6 %)

^a^ Response to the question: “In any one year, have you had at least 12 drinks of any type of alcoholic beverage?”

^b^ Response to the question: “In a typical week do you do any moderate-intensity sports, fitness, or recreational activities that cause a small increase in breathing or heart rate such as brisk walking, bicycling, swimming, or volleyball for at least 10 minutes continuously?”

^c^ Defined as a history of heart failure, coronary heart disease, or angina

Table 2 shows the distribution of participants among cognitive function tests, and the means, medians and Pearson correlation coefficients among log-transformed sodium levels and cognitive test scores. The geometric mean of log-transformed serum sodium was 4.938 (± 0.0008) mmol/L, and the median (interquartile range) scores for the CERAD WLT-IR, CERAD WLT-DR, AFT, and DSST were 19 (16-22), 6 (4-8), 16 (13-20), and 46 (34–59) respectively. Sodium showed positive correlations with the test scores, and all correlations were significant excluding the relationship between sodium and AFT score, which showed borderline significance. Statistically significant (p < 0.05) positive correlation coefficients were observed between all cognitive test scores.

**Table 2:** Means, medians and Pearson correlation coefficients among log-transformed sodium levels and cognitive test scores in the study population

	n	Geometric mean (SE) or Median (IQR)	Pearson’s correlation coefficients (p-value)				
			**Sodium, mmol/L**	**CERAD WLT**		**AFT score**	**DSST score**
				**IR score**	**DR score**		
**Sodium, mmol/L**		4.938 (0.0008)	1.00				
**CERAD WLT-IR score**	2541	19 (16–22)	0.10 (< 0.001)	1.00			
**CERAD WLT-DR score**	2540	6 (4–8)	0.08 (< 0.001)	0.73 (< 0.001)	1.00		
**AFT score**	2523	16 (13–20)	0.04 (0.068)	0.39 (< 0.001)	0.35 (< 0.001)	1.00	
**DSST score**	2465	46 (34–59)	0.07 (0.001)	0.47 (< 0.001)	0.45 (< 0.001)	0.50 (< 0.001)	1.00

Table 3 shows the results of linear regression analyses between log-transformed serum sodium and quintiles of cognitive test scores. CERAD WLT-IR and CERAD WLT-DR showed significant positive associations with log-transformed sodium levels in all models (CERAD WLT-IR: unadjusted model: 5.35 (SE = 1.88, p-value 0.008), adjusted model: 4.25 (SE = 1.83, p-value 0.027); CERAD WLT-DR: unadjusted model: 6.91 (SE = 1.95, p-value 0.001), adjusted model: 6.54 (SE = 1.82, p-value 0.001)). Beta coefficients for the associations between AFT and DSST scores, and sodium levels showed positive associations, but were not statistically significant.

**Table 3:** Associations between log-transformed serum sodium levels and quintiles of cognitive test scores by linear regression models

**Model**	**CERAD WLT-IR (n=2541)**		**CERAD WLT-DR (n=2540)**		**AFT (n=2523)**		**DSST (n=2465)**	
	**Beta ± SE**	**P**	**Beta ± SE**	**P**	**Beta ± SE**	**P**	**Beta ± SE**	**P**
**Unadjusted**	5.35 **± **1.88	0.008	6.91 **± **1.95	0.001	2.35 **± **1.53	0.136	2.92 **± **1.88	0.129
**Adjusted**^a^	4.25 **± **1.83	0.027	6.54 **± **1.82	0.001	2.12 **± **1.39	0.135	1.59 **± **1.64	0.341

^a^ Adjusted for sex, age, marital status, ethnicity, smoking status, alcohol consumption, physical activity, income, obesity, education, GFR, serum glucose levels, diuretics use, diabetes, CVD, stroke, COPD, thyroid disease, and liver disease

Table 4 shows the associations between log-transformed serum sodium levels and cognitive test scores by hyponatremia and hypernatremia status. Compared to normal sodium levels, hyponatremia was significantly associated with lower cognitive test scores in the unadjusted model (CERAD WLT-IR beta = -0.58, SE = 0.18, p-value 0.002; CERAD WLT-DR beta = -0.69, SE = 0.15, p-value < 0.001; AFT beta = -0.62, SE = 0.19, p-value 0.002; DSST beta = -0.52, SE = 0.23, p-value 0.030). In the adjusted model, hyponatremia was significantly associated with lower CERAD WLT-IR (beta = -0.34, SE = 0.15, p-value 0.035) and CERAD WLT-DR scores (beta = -0.48, SE = 0.10, p-value < 0.001), and showed borderline significance with AFT scores (beta = -0.38, SE = 0.19, p-value 0.052), compared to normal sodium levels. In contrast, the hypernatremia group did not show any significant relationships with cognitive test scores, compared to the normal sodium group.

Linear regression analysis of quintiles of cognitive function among patients with normal sodium levels and low sodium levels, did not show any significant linear relationships, excluding the adjusted relationship between log-transformed normal serum sodium levels and quintiles of CERAD WLT-DR (beta = 5.61, SE= 3.03, p-value 0.074), which showed borderline significance, as shown in Table 5.

**Table 4:** Linear regression analysis of log-transformed serum sodium levels and quintiles of cognitive test scores by hyponatremia and hypernatremia status

	**n**	**Unadjusted model**		**Adjusted model**^a^	
		**Beta ± SE**	**P**	**Beta ± SE**	**P**
**CEARD WLT-IR**					
Normal	2446	1		1	
Hyponatremia	87	-0.58 **± **0.18	0.002	-0.34 **± **0.15	0.035
Hypernatremia	8	0.39 **± **0.67	0.565	0.13 **± **0.68	0.852
**CEARD WLT-DR**					
Normal	2445	1		1	
Hyponatremia	87	-0.69 **± **0.15	<0.001	-0.48 **± **0.10	<0.001
Hypernatremia	8	0.60 **± **0.59	0.318	0.42 **± **0.62	0.496
**AFT**					
Normal	2428	1		1	
Hyponatremia	86	-0.62 **± **0.19	0.002	-0.38 **± **0.19	0.052
Hypernatremia	9	-0.63 **± **0.65	0.334	-0.69 **± **0.44	0.127
**DSST**					
Normal	2376	Reference		Reference	
Hyponatremia	81	-0.52 **± **0.23	0.030	-0.18 **± **0.21	0.382
Hypernatremia	8	0.23 **± **0.47	0.620	0.07 **± **0.41	0.866

^a^Adjusted for sex, age, marital status, ethnicity, smoking status, alcohol consumption, physical activity, income, obesity, education, GFR, serum glucose levels, diuretics use, diabetes, CVD, stroke, COPD, thyroid disease, and liver disease

**Table 5:** Linear regression analysis of log transformed serum sodium levels and quintiles of cognitive test scores stratified by serum sodium levels

**Model**	**CERAD WLT-IR**		**CERAD WLT-DR**		**AFT**		**DSST**	
	**Beta ± SE**	**P**	**Beta ± SE**	**P**	**Beta ± SE**	**P**	**Beta ± SE**	**P**
Hyponatremia (<135 mmol/L)								
n	87		87		86		81	
Unadjusted	5.36 **± **12.62	0.674	3.17 **± **12.43	0.800	3.57 **± **11.78	0.764	9.84 **± **10.99	0.379
Model 1^a^	17.51 **± **12.67	0.179	15.96 **± **11.08	0.162	-1.39 **± **8.56	0.873	2.74 **± **10.12	0.789
Normal serum sodium (135-145 mmol/L)								
n	2446		2445		2428		2376	
Unadjusted	2.50 **± **3.04	0.418	4.35 **± **3.06	0.164	-1.20 **± **2.08	0.569	-1.03 **± **2.13	0.632
Model 1^a^	3.16 **± **2.80	0.267	5.61 **± **3.03	0.074	1.08 **± **2.19	0.625	0.22 **± **1.75	0.900

^a^ Adjusted for sex, age, marital status, ethnicity, smoking status, alcohol consumption, physical activity, income, obesity, education, GFR, serum glucose levels, diabetes, CVD, COPD, thyroid disease, and liver disease

## Discussion

In a cross-sectional study of the United States population sampled from the 2010–2011 and 2013–2014 NHANES, serum sodium levels were positively associated with cognitive test scores in the elderly. Log-transformed serum sodium levels were significantly associated with quintiles of CERAD WLT scores. In addition, compared to normal sodium levels, hyponatremia was associated with lower CERAD WLT (memory sub-domain) and AFT (executive function) scores. In short, serum sodium levels were significantly associated with the memory sub-domain assessed by the CERAD WLT and executive function assessed by the AFT, but not with processing speed, sustained attention, and working memory evaluated by the DSST.

In line with the results of our study, previous studies have reported an association between low sodium levels and cognitive function; however, these studies have only examined single domains of cognitive function (i.e. attention), or have assessed multiple domains grouped as a single variable. A case-control study performed in a general hospital in Brussels reported that individuals with low serum sodium levels (n = 122, range: 115–132 mmol/L, mean ± standard deviation [SD] = 126 ± 5 mmol/L) were associated with a higher odds of falling (odds ratio [OR] = 67.43, 95 % confidence interval [CI] = 7.48–607.42) and significantly slower mean response times in the attention tests (difference: 58 milliseconds, P < 0.001) compared to individuals with normal sodium levels (n = 244, mean ± SD = 139 ± 2 mmol/L) [7]. Eight different visual and auditory attention tests were included in the battery of attentional tests [7]. A cross-sectional study from the Hunter Community Study, a population-based prospective cohort study in Australia, revealed that standardized ARCS scores were significantly higher (mean = 4.67 units, 95 % CI = 1.56–7.79, p = 0.01) for subjects with serum sodium levels equal to 135 mmol/L than those with serum sodium levels of 130 mmol/L [8]. The ARCS scores is a vague tool which reflects a wide range of cognitive domains [23]. A recent study from the Osteoporotic Fractures in Men study, revealed that men with sodium levels of 126–140 mmol/L were associated with a 1.30 odds (95 % CI = 1.06–1.61) of cognitive impairment, compared to individuals with sodium levels of 141–142 mmol/L. In this study, cognitive impairment was defined as a modified Mini-Mental Status score less than 84 or a Trail Making Test Part B time greater than 233 s [9]. In addition, a study conducted in 150 patients aged 70 years and older from the University Hospital Cologne, showed that resolution of hyponatremia (< 130 mmol/L) by > 5 mmol/L was significantly associated with an increase in MMSE scores (ΔMMSE: 1.8 ± 3.0 vs. 0.7 ± 1.9; p = 0.002) [24]. Reversibility has also been reported in previous animal studies mentioned below [25, 26].

The mechanisms behind this association is unclear; however, abnormalities in brain osmolyte levels such as glutamate may play a role [6, 25, 26]. In chronic hyponatremia, brain cells export osmolytes such as glutamate, which may influence memory [27, 28]. An animal study showed that sustained low levels of serum sodium concentrations caused reversible cognitive impairment measured by a novel object recognition test and contextual fear conditioning tests [26]. In vivo analysis of brain samples from chronic hyponatremic rats in this study revealed elevated extracellular glutamate concentrations in the hippocampus and decreased glutamate uptake by astrocyte cultures [26, 29]. In addition, another study in rats proposed brain cell swelling as another explanation. In this study, rats with chronic hyponatremia, which showed reversible memory impairments assessed using the passive avoidance test, also showed brain swelling [25]. However, the reason why lower serum sodium levels are associated with memory sub-domain and executive function, in contrast to sustained attention, working memory, and processing speed, is unknown, and further studies are recommended to clarify the mechanism behind such association.

Our study has examined the relationship between serum sodium levels and various cognitive domains in a nation-wide population of US adults. Nevertheless, our study has several limitations. Firstly, due to the cross-sectional nature of the study, any casual or temporal relationships cannot be assessed. Moreover, due to the cross-sectional nature of the NHANES data, we were unable to differentiate between acute and chronic hyponatremia. Despite this fact, it is more likely that the sodium status of most participants reflect chronic levels as acute changes in sodium levels are known to cause neurologic symptoms (i.e. seizures and altered mental status) as a result of cerebral edema [3]. Thirdly, the relationship between cognitive test scores and serum sodium levels may not necessarily be linear. Resultantly, our results may not be generalized to populations with different sodium levels. Fourthly, although we have considered basic sociodemographic variables, GFR, and serum glucose levels as covariates, previous studies have also included quality-of-life measures [7, 9]. Therefore, we cannot completely eliminate the possibility of residual confounding. Fifthly, the range of cognitive test scores performed by the NHANES remains limited, and further studies evaluating the relationship between other domains of cognitive function and serum sodium levels are recommended. Additionally, among a total of 3,632 individuals aged 60 years and older who participated in the 2011–2014 NHANES, only 2,541 for CERAD WLT-IR, 2,540 for CERAD WLT-DR, 2,523 for AFT, and 2,465 for DSST were included in the final analysis due to various reasons (refusal, language/communication problems, failure to pass the practice test, need for a proxy, time constrains, and missing data on serum sodium or covariates). Because cognitive test scores were missing for subjects who needed a proxy, did not pass the practice test, and quit or gave up, the overall study sample may have included those with relatively higher cognitive function scores, limiting the external validity of our results. Lastly, individuals with hypernatremia may have shown no association due to the small sample sizes (< 10). Further studies should be conducted with larger sample sizes to investigate the relationship between hypernatremia and the separate domains of cognitive function.

## Conclusions

In conclusion, lower sodium levels were associated with cognitive change, especially regarding memory and executive function in a population based study of US adults aged 60 and over, pointing to hyponatremia as a risk factor of cognitive impairment. When evaluating memory and executive function in the elderly, serum sodium levels should be taken into account, as they may cause reversible changes in cognitive function. In addition, further studies should be conducted in order to elucidate the mechanism behind such relationships.

## Data Availability

The datasets generated and/or analyzed during the current study are available on the NHANES website, https://www.cdc.gov/nchs/nhanes/index.htm.

## Abbreviations

CERAD WLT: CERAD Word Learning Test
IR: Immediate recall
DR: Delayed recall
DSST: Digit Symbol Substitution test
AFT: Animal Fluency test
NHANES: National Health and Nutrition Examination Surveys
BMI: Body mass index
GFR: Glomerular filtration rate
CKD-EPI eGFR: Chronic Kidney Disease Epidemiology Collaboration equation
COPD: Chronic obstructive pulmonary disease
SE: Standard errors
OR: Odds ratio
SD: Standard deviation
CI: Confidence interval
ARCS: Audio Recorded Cognitive Screening
MMSE: Mini-mental state examination

## References

[1] Shrimanker I, Bhattarai S. Electrolytes. StatPearls. Treasure Island (FL): StatPearls Publishing. Copyright © 2020, StatPearls Publishing LLC.; 2020.

[2] Terry J (1994). The major electrolytes: sodium, potassium, and chloride. Journal of intravenous nursing: the official publication of the Intravenous Nurses Society.

[3] Sahay M, Sahay R, Hyponatremia (2014). A practical approach. Indian J Endocrinol Metab.

[4] Upadhyay A, Jaber BL, Madias NE (2006). Incidence and prevalence of hyponatremia. Am J Med.

[5] Gosch M, Joosten-Gstrein B, Heppner HJ, Lechleitner M (2012). Hyponatremia in Geriatric Inhospital Patients: Effects on Results of a Comprehensive Geriatric Assessment. Gerontology.

[6] Soiza RL, Cumming K, Clarke JM, Wood KM, Myint PK (2014). Hyponatremia: Special Considerations in Older Patients. Journal of clinical medicine.

[7] Renneboog B, Musch W, Vandemergel X, Manto MU, Decaux G (2006). Mild chronic hyponatremia is associated with falls, unsteadiness, and attention deficits. Am J Med.

[8] Gunathilake R, Oldmeadow C, McEvoy M, Kelly B, Inder K, Schofield P (2013). Mild hyponatremia is associated with impaired cognition and falls in community-dwelling older persons. J Am Geriatr Soc.

[9] Nowak KL, Yaffe K, Orwoll ES, Ix JH, You Z, Barrett-Connor E (2018). Serum Sodium and Cognition in Older Community-Dwelling Men. Clinical journal of the American Society of Nephrology: CJASN.

[10] Harada CN, Natelson Love MC, Triebel KL (2013). Normal cognitive aging. Clin Geriatr Med.

[11] Wisdom NM, Mignogna J, Collins RL (2012). Variability in Wechsler Adult Intelligence Scale-IV subtest performance across age. Archives of clinical neuropsychology: the official journal of the National Academy of Neuropsychologists.

[12] Babcock H (1930). An experiment in the measurement of mental deterioration. Archives of Psychology.

[13] Harvey PD (2019). Domains of cognition and their assessment. Dialogues Clin Neurosci.

[14] U. S. Centers for Disease Control Prevention. National health and nutrition examination survey 2019 [Available from: https://www.Cdc.Gov/nchs/nhanes/index.Htm. Accessed 2 May 2021.

[15] U. S. Centers for Disease Control Prevention. 2013–2014 Data Documentation, Codebook, and Frequencies: Cognitive Functioning (CFQ_H) 2017 [Available from: https://wwwn.cdc.gov/Nchs/Nhanes/2013-2014/CFQ_H.htm. Accessed 2 May 2021.

[16] U. S. Centers for Disease Control Prevention. 2011–2012 Data Documentation, Codebook, and Frequencies: Cognitive Functioning (CFQ_G) 2017 [Available from: https://wwwn.cdc.gov/Nchs/Nhanes/2011-2012/CFQ_G.htm. Accessed 2 May 2021.

[17] U. S. Centers for Disease Control Prevention. NHANES 2013–2014 Laboratory Data Overview [updated February 21 2020. Available from: https://wwwn.cdc.gov/nchs/nhanes/ContinuousNhanes/OverviewLab.aspx?BeginYear=2013. Accessed 2 May 2021.

[18] U. S. Centers for Disease Control Prevention. NHANES 2011–2012 Laboratory Data Overview [updated February 21 2020. Available from: https://wwwn.cdc.gov/nchs/nhanes/ContinuousNhanes/OverviewLab.aspx?BeginYear=2011. Accessed 2 May 2021.

[19] U. S. Centers for Disease Control Prevention. 2011–2012 Data Documentation, Codebook, and Frequencies: Standard Biochemistry Profile (BIOPRO_G) 2013 [updated Feb 2014. Available from: https://wwwn.cdc.gov/Nchs/Nhanes/2011-2012/BIOPRO_G.htm. Accessed 2 May 2021.

[20] U. S. Centers for Disease Control Prevention. 2013–2014 Data Documentation, Codebook, and Frequencies: Standard Biochemistry Profile (BIOPRO_H) 2015 [Available from: https://wwwn.cdc.gov/Nchs/Nhanes/2013-2014/BIOPRO_H.htm. Accessed 2 May 2021.

[21] Levey AS, Stevens LA, Schmid CH, Zhang YL, Castro AF, Feldman HI (2009). A new equation to estimate glomerular filtration rate. Ann Intern Med.

[22] U. S. Centers for Disease Control Prevention. Tutorials Module 3: Weighting [updated August 4 2020. Available from: https://wwwn.cdc.gov/nchs/nhanes/tutorials/module3.aspx. Accessed 2 May 2021.

[23] Schofield PW, Lee SJ, Lewin TJ, Lyall G, Moyle J, Attia J (2010). The Audio Recorded Cognitive Screen (ARCS): a flexible hybrid cognitive test instrument. J Neurol Neurosurg Psychiatry.

[24] Brinkkoetter PT, Grundmann F, Ghassabeh PJ, Becker I, Johnsen M, Suaréz V (2019). Impact of Resolution of Hyponatremia on Neurocognitive and Motor Performance in Geriatric Patients. Sci Rep.

[25] Miyazaki T, Ohmoto K, Hirose T, Fujiki H (2010). Chronic hyponatremia impairs memory in rats: effects of vasopressin antagonist tolvaptan. J Endocrinol.

[26] Fujisawa H, Sugimura Y, Takagi H, Mizoguchi H, Takeuchi H, Izumida H (2016). Chronic Hyponatremia Causes Neurologic and Psychologic Impairments. J Am Soc Nephrol.

[27] McEntee WJ, Crook TH (1993). Glutamate: its role in learning, memory, and the aging brain. Psychopharmacology.

[28] Massieu L, Montiel T, Robles G, Quesada O (2004). Brain amino acids during hyponatremia in vivo: clinical observations and experimental studies. Neurochem Res.

[29] Anderson CM, Swanson RA (2000). Astrocyte glutamate transport: review of properties, regulation, and physiological functions. Glia.

